# Diagnosis of hereditary transthyretin amyloidosis in patients with suspected chronic inflammatory demyelinating polyneuropathy unresponsive to intravenous immunoglobulins: results of a retrospective study

**DOI:** 10.1186/s13023-025-03589-4

**Published:** 2025-03-01

**Authors:** Yann Péréon, David Adams, Jean-Philippe Camdessanché, Jean-Baptiste Chanson, Pascal Cintas, Laurent Magy, Aïssatou Signaté, Guilhem Solé, Juliette Svahn, Céline Tard, Cyrla Hababou, Shahram Attarian

**Affiliations:** 1 Centre de Référence Maladies Neuromusculaires AOC, Filnemus, Euro-NMD, Hôtel-Dieu, CHU de Nantes, Nantes, France; 2 Département de Neurologie, Centre de Référence Neuropathies Rares CERAMIC, CHU de Bicêtre, Université Paris-Saclay, Paris, France; 3 Department of Neurology, Reference Centre for Neuromuscular Diseases, Hôpital Nord, University Hospital of Saint-Etienne, Saint-Etienne, France; 4 Service de Neurologie, Hôpitaux Universitaires de Strasbourg et Centre de Référence Neuromusculaire, Nord/Est/Ile de France, Strasbourg, France; 5 Hôpital Pierre Paul Riquet, Centre de Référence de Pathologie Neuromusculaire, Toulouse, France; 6 Service et Laboratoire de Neurologie, Centre de Référence National Neuropathies Périphériques Rares, Centre Hospitalier Universitaire Dupuytren, Limoges, France; 7 Service de Neurologie, Centre Hospitalier Universitaire de Martinique, Fort-De-France, France; 8 Centre de Référence des Maladies Neuromusculaires AOC, Service de Neurologie et des Maladies Neuromusculaires, Hôpital Pellegrin, Centre Hospitalier Universitaire de Bordeaux, Filnemus, Euro-NMD, Bordeaux, France; 9 Service de Pathologies Neuromusculaires, Hôpital Pierre Wertheimer, Hospices Civils de Lyon, Lyon, France; 10 Service de Neurologie, CHU de Lille, Centre de Référence des Maladies Neuromusculaires Nord/Est/Ile-de-France, Lille, France; 11 Laboratoire Alnylam, 100 avenue de Suffren, Paris, 15015 France; 12 Centre de Référence des Maladies Neuromusculaires et de la SLA, CHU la Timone, Aix-Marseille Université, Marseille, France

**Keywords:** Chronic inflammatory demyelinating polyradiculoneuropathy, Hereditary transthyretin amyloidosis, Intravenous immunoglobulins

## Abstract

**Background and aims:**

Hereditary transthyretin amyloidosis (ATTRv) should be considered in patients diagnosed with intravenous immunoglobulin (IVIg)-resistant chronic inflammatory demyelinating polyradiculoneuropathy (IVIg-NR CIDP). In this 1-year long, retrospective, multicentric study, an online questionnaire was sent to 1100 French healthcare professionals (HCPs) investigating: (i) how many IVIg-NR CIDP patients they followed; (ii) how many IVIg-NR CIDP patients had undergone *TTR* gene analysis; and (iii) how many IVIg-NR CIDP patients were eventually diagnosed with ATTRv. The questionnaire was sent every 3 months for 1 year and contained information on ATTRv clinical manifestations and diagnosis.

**Results:**

One-hundred and ten (10%) HCPs responded. A total of 2131 patients with CIDP were identified, including 315 (22.1%) with IVIg-NR CIDP. *TTR* gene analysis was performed in 144 patients and was positive in 43 cases (29.9%).

**Conclusions:**

This study demonstrates that ATTRv should be investigated systematically in patients diagnosed with IVIg-NR CIDP. HCP-directed information campaigns are useful for modifying diagnostic practices.

**Supplementary Information:**

The online version contains supplementary material available at 10.1186/s13023-025-03589-4.

## Background

Chronic inflammatory demyelinating-like polyradiculoneuropathy (CIDP) is an autoimmune disease of the peripheral nervous system affecting myelin. CIDP is rare, with a reported prevalence ranging from 0.15 to 8.9 cases/100 000 individuals [1–3] and may present at any age. The typical form is characterized by a symmetrical proximal-distal sensorimotor deficit of the upper and lower limbs with gradual onset over at least 8 weeks [1]. The clinical course of CIDP varies and can be either progressive or relapsing-remitting.

The diagnosis of CIDP is based on a combination of clinical, electrophysiological, biological and paraclinical data [4, 5]. According to the 2021 criteria of the European Academy of Neurology/Peripheral Nerve Society (EAN/PNS), the spectrum of CIDP now includes typical CIDP and four variants: distal, multifocal/focal, motor and sensory CIDP [4]. CIDP variants are more difficult to diagnose, especially focal and multifocal variants, leading to diagnostic delays [4, 6]. In an attempt to simplify the diagnosis, the levels of diagnostic certainty have been reduced from three (definite, probable, possible CIDP) to two (CIDP and possible CIDP) since there was no difference in the diagnostic accuracy of criteria for probable and definite CIDP [4]. 

The majority of patients with CIDP are treated with corticosteroids, high-dose intravenous immunoglobulins (IVIg) or plasma exchanges [2, 4, 6]. Clinical improvement after immune therapy is a useful parameter to confirm the diagnosis. The recent, updated consensus guidelines of the EAN/PNS recommend IVIg or corticosteroids as first-line treatment for typical and CIDP variants [4]. Induction treatment duration is 4‒6 weeks, with maintenance treatment necessary at regular time intervals for many years [4]. 

In a recent retrospective chart review, Godil et al. reported that 38% (15/40) of their patients with CIDP were not responsive to standard IVIg treatment (IVIg-NR) [7]. The incidence of IVIg-NR CIDP was 9-times higher in patients with distal CIDP than in those with other CIDP variants [7]. Resistance to IVIg should question CIDP diagnosis and suggest the possibility of other diseases such as hereditary transthyretin amyloidosis (ATTRv), Polyneuropathy- Organomegaly-Endocrinopathy-M gammopathy-Skin changes (POEMS) syndrome, vasculitis, neurolymphoma.

The incidence of ATTRv is estimated to be 13–15% among patients with possible CIDP who do not respond to IVIg [8]. This is a rare, autosomal dominant, adult-onset, treatable disease, which usually has a late onset, developing after the age of 50 years in 75% of patients and around 30 years of age in 25%, with geographical variations. Although uncommon, the disease is being diagnosed with increasing frequency worldwide [9]. It is caused by variants in the transthyretin (*TTR*) gene resulting in hepatic secretion of a mutated TTR protein, which accumulates in organs, primarily in the peripheral nerves, heart, digestive tract and eyes, in the form of fibrillar deposits, known as amyloid deposits [10, 11]. Over 130 *TTR* gene mutations have been identified [9]. 

The clinical manifestations of ATTRv include sensation loss and walking difficulties, but also weight loss, digestive, sexual, kidney, eye and heart disorders [10], leading to a loss of autonomy and death within an average of 10 years without treatment [11]. 

ATTRv underdiagnosis and misdiagnosis are the main barriers to initiating effective treatment, since these therapies are most effective when they are started in the early stages of the disease [12]. Once the disease is suspected, the gold-standard diagnostic procedure is based on *TTR* gene analysis [13, 14]. In non-endemic areas, and in the presence of a negative family history, the diagnosis may be delayed by 4‒5 years [14–16]. 

The aim of this survey, carried out among healthcare professionals (HCPs) across France, was to determine the percentage of patients with suspected CIDP who were IVIg-NR and subsequently diagnosed with ATTRv, including the percentage of IVIg-NR patients who underwent *TTR* genetic testing.

## Methods

### Study design and study population

This retrospective study was carried out over a 1-year period between December 2020 and December 2021.

An online questionnaire (Appendix) was sent to 1100 HCPs across France with either a private or public practice. These HCPs were selected as they care for the majority of patients with CIDP in France. The HCPs were recruited by the independent company EXAFIELD, which specializes in the recruitment of clinical personnel for market research. All participants were remunerated for taking part in the study.

The HCPs were contacted by telephone and after validation of the qualification criteria, a secure connection link was sent by e-mail to the respondent to answer the questionnaire. The HCPs were asked a number of questions including how they managed and diagnosed their CIDP patients, how many patients with suspected CIDP they had, how many patients were treated with IVIg and were IVIg-NR, how many had undergone *TTR* gene analysis, and how many had tested positive (Appendix). IVIg-NR was defined as no response to IVIg after 3–4 months of treatment. Only patients with probable or definite CIDP using the 2010 EFNS/PNS diagnostic criteria [17] were included in the survey. The questionnaires were sent to the participants every 3 months for 1 year. The questionnaires were identical in each of the four waves of the survey.

The aim of the study was to determine the percentage of patients with suspected CIDP who were IVIg-NR and subsequently diagnosed with ATTRv.

## Results

### Study population

**Fig. 1:**
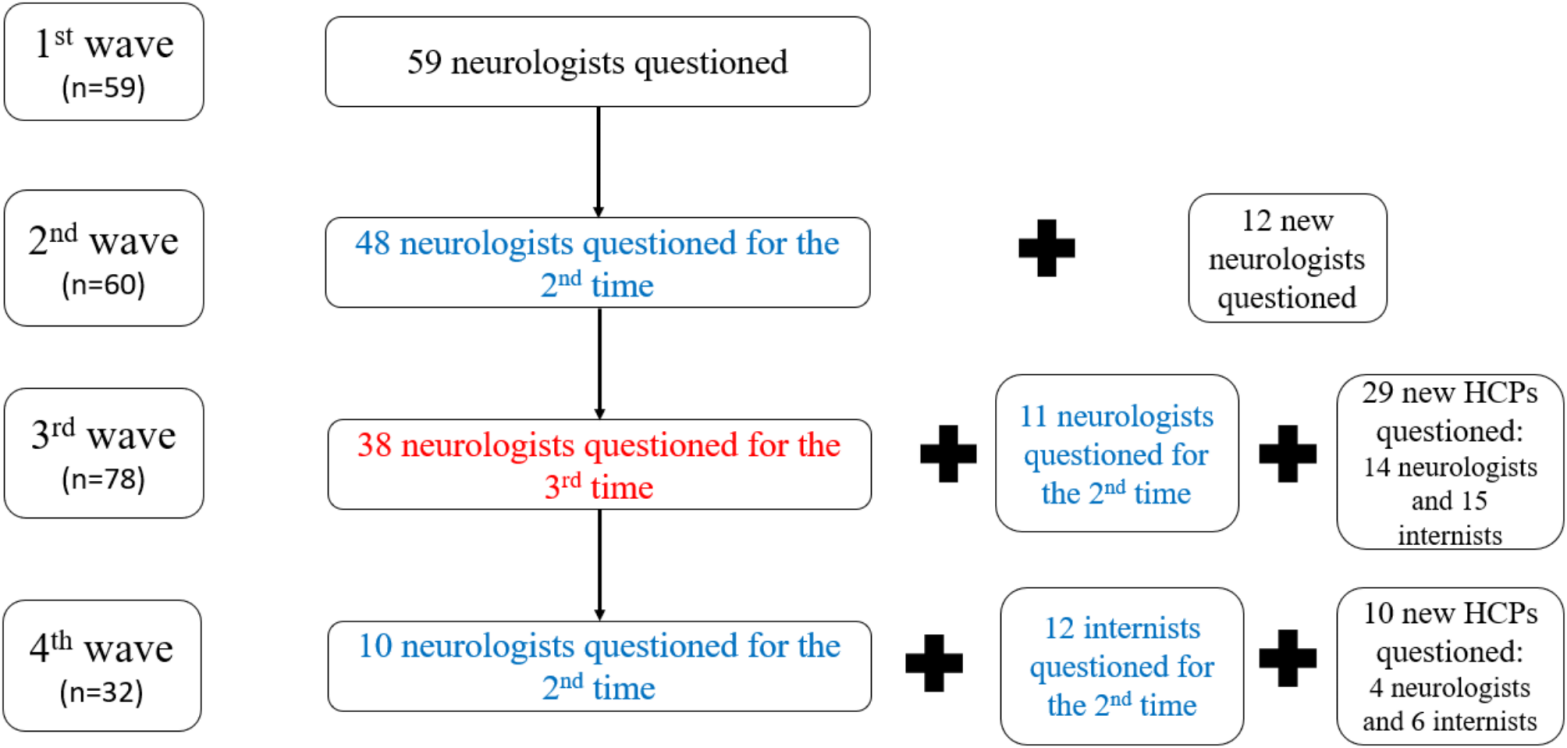
Number of healthcare professionals questioned in each wave of the survey

A total of 110/1100 (10%) HCPs (mean (± SD) age: 46.0 ± 8.8 years; 64% male) responded to at least one wave of the survey. There were 59 new respondents in the first wave, 12 in the second, 29 in the third and 10 in the fourth (Fig. 1). Eighty HCPs responded to two waves and 38 responded to three waves of the survey. The distribution of the respondents in each wave of the survey is shown in Fig. 1. The respondents were well distributed across France.

### *TTR* gene analysis in patients with suspected CIDP who were IVIg-NR

**Fig. 2:**
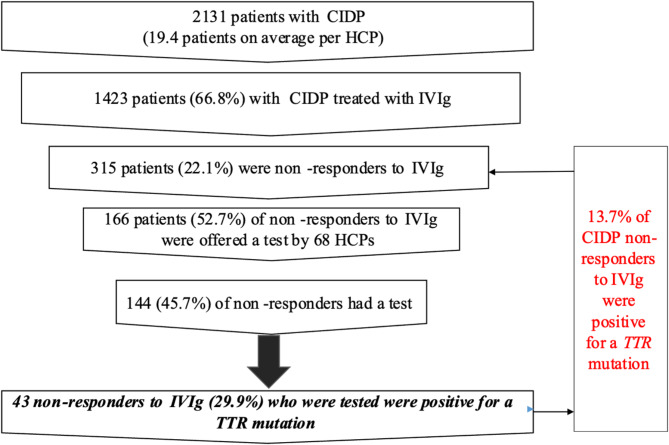
Results for healthcare professionals who responded at least once to the survey

A total of 2131 patients with suspected CIDP were identified (mean of 19.4 patients per HCP). The HCPs were not asked about which variant of CIDP the patients had been diagnosed with. Two-thirds of these patients (*n* = 1423; 66.8%) were treated with IVIg, of which 315 (22.1%) were IVIg-NR. A *TTR* test was proposed to 166 IVIg-NR patients (52.7%) and 144 (45.7%) underwent a test. Forty-three of these (29.9%) tested positive for a *TTR* pathogenic variant. Overall, 13.7% of IVIg-NR patients with suspected CIDP tested positive for such variants. These results are summarized in Fig. 2.

### *TTR* gene analysis in IVIg-NR patients according to the number of times HCPs were exposed to the survey

Among the HCPs who responded to the survey at least once, 67.5% (966/1431) of their patients were treated with IVIg. One-hundred and ninety-eight (20.5%) of these patients were IVIg-NR and 93 (47%) were proposed *TTR* testing. Seventy-five of these 93 patients (80.0%) underwent the test and 23 (30.7%) were positive.

For the 80 HCPs who responded to the survey twice, 57.9% (283/489) of their new patients were treated with IVIg, 76 (19.8%) of these patients were IVIg-NR and 46 (60.5%) were proposed *TTR* testing. Forty-two of these 46 patients (91.3%) underwent the test and 16 (38.0%) were positive.

Finally, for the 38 HCPs who responded to the survey three times, 174/211 (82.5%) new patients were treated with IVIg and 41/174 (23.6%) were IVIg-resistant. Twenty-seven (65.9%) were proposed *TTR* testing and all (100%) underwent it. Four of these patients (14.8%) were positive. These results are summarized in Table 1.

**Table 1:** Comparison of the results in the different waves of the survey

	One survey exposure	Two survey exposures	Three survey exposures
No. of HCPs	110	80	38
No. of new suspected CIDP patients	1431	489	211
No. of patients with suspected CIDP treated with IVIg	966 (67.5%)	383 (78.3%)	174 (82.5%)
No. of patients who were IVIg-NR	198 (20.5% of IVIg treated patients)	76 (19.8% of IVIg treated patients)	41 (23.6% of IVIg treated patients)
No. of IVIg-NR patients proposed a *TTR* test	93 (47.0% of non-responders)	46 (60.5% of non-responders)	27 (65.9% of non-responders)
No. of IVIg-NR patients where a *TTR* test was performed	75 (37.9% of non-responders)	42 (55.3% of non-responders)	27 (65.9% of non-responders)
No. of IVIg-NR patients who were *TTR* test positive	23 (30.7% of tests performed). 11.6% of IVIg-NR CIDP patients	16 (38.1% of tests performed) 21.0% of IVIg-NR CIDP patients	4 (14.8% of tests performed). 9.7% of IVIg-NR CIDP patients

### Reasons for prescribing or not prescribing *TTR* gene analysis

A small population of HCPs indicated the reasons why they did (*n* = 13) or did not (*n* = 17) prescribe *TTR* testing. The most frequent reason for prescribing *TTR* gene analysis was orthostatic hypotension (69%) (Table 2) while the most common reasons for not offering a *TTR* test were an alternative diagnosis (41%) or a lack of knowledge about the differential diagnosis of CIDP (41%) (Table 3).

**Table 2:** Main reasons why a *TTR* test was proposed to the IVIg-NR suspected CIDP patients

Reason a TTR test was proposed	HCPs (*n* = 13)
**Orthostatic hypotension**	9 (69%)
**Upper limb weakness**	6 (46%)
**Gastrointestinal disorders**	6 (46%)
**Proteinuria**	5 (38%)
**Erectile dysfunction**	4 (31%)
**Unexplained weight loss**	4 (31%)
**Pain**	1 (8%)
**Glaucoma**	1 (8%)

**Table 3:** Main reasons why a *TTR* test was not proposed to the IVIg-NR suspected CIDP patients

Reason a TTR test was not proposed or performed	HCPs (*n* = 17)
**Alternative diagnosis**	7 (41%)
**Lack of information about the differential diagnosis of CIDP**	7 (41%)
**Lack of information on where and how to carry out a test**	3 (18%)
**Patient refusal**	1 (12%)
**Other reason**	2 (12%)

## Discussion

The aim of this survey was to determine the percentage of patients with suspected CIDP who were IVIg-NR and were subsequently diagnosed with ATTRv, including the percentage of IVIg-NR patients who underwent *TTR* genetic testing.

Patients with CIDP experience muscle weakness of the upper and lower limbs, and sensory disturbances [5]. Five subgroups of CIDP have been identified, namely typical CIDP and CIDP variants (distal, multifocal/focal, motor and sensory CIDP) [4, 5]. Some features are common to all forms of CIDP, including gradual disease onset over a period of > 8 weeks, areflexia, nerve changes indicative of segmental demyelination and, generally, a positive response to immunomodulation with IVIg, corticosteroids or plasma exchanges [5]. However, the clinical presentation may differ between CIDP subgroups and a number of red flags have been raised that are suggestive of possible alternative diagnoses. These have been summarized very succinctly by Lexis et al. [5]. based on the 2021 EAN/PAS guidelines [4]. The differential diagnoses for typical CIDP includes Guillain-Barré syndrome, multiple myeloma, osteosclerotic myeloma, POEMS syndrome, amyloid light-chain amyloidosis, ATTRv, HIV-related polyneuropathy, hepatic polyneuropathy, uraemic polyneuropathy, vitamin B12 deficiency, autoimmune nodopathy and CANOMAD (chronic neuropathy ophthalmoplegia M-protein agglutination disialosyl antibodies), with further differential diagnoses for CIDP variants [5]. These authors [5] also summarized the clinical and electrophysiological criteria for the diagnosis of CIDP and its variants based on the 2021 EAN/PAS guidelines [4], with a flow chart indicating the key steps in the differential diagnosis of typical and variant CIDP [5]. Our patients were diagnosed with suspected CIDP using the 2010 EFNS/PNS diagnostic criteria [17]. A patient was considered to be IVIg-NR if they had no response to IVIg after 3–4 months of treatment.

IVIg-resistant CIDP is one of the main differential diagnoses for ATTRv, with the incidence of hATTR estimated at 13–15% among IVIg-NR CIDP patients [8]. This was confirmed in our study where 13.7% of IVIg-NR suspected CIDP patients tested positive for a *TTR* mutation. However, in the current survey, despite the fact that around 22% of patients with suspected CIDP treated with IVIg were non-responders, over half (54.3%) of these IVIg-NR patients did not undergo *TTR* testing. It is therefore possible that some patients with ATTRv could have been missed.

Our results also show that the rate of *TTR* testing increased the more times a HCP was exposed to and answered the survey, from 37.9% for HCPs who answered the survey once to 65.9% for those that answered it three times. Thus, being asked the question about the genetic test for ATTRv (Appendix) seemed to prompt more HCPs to get their patients tested.

In our study, the most common reason for referring a patient for *TTR* testing after a lack of response to IVIg was dysautonomia, manifest as orthostatic hypotension, gastrointestinal problems and erectile dysfunction, which is evocative of ATTRv. Orthostatic hypotension, defined as a decrease of at least 20 mmHg in systolic blood pressure or 10 mmHg in diastolic blood pressure within 3 min of standing or tilting upright [18], is a common manifestation of dysautonomia in ATTRv and affects between 40 and 60% of patients [19]. Carpal tunnel syndrome associated with systemic symptoms (dysautonomia, including erectile dysfunction, gastrointestinal polyneuropathy, exercise intolerance, and ocular symptoms) has also been shown to be an early indicator of ATTRv [20], although this syndrome was not included in our questionnaire.

The prognosis of patients with ATTRv has improved significantly over recent years with a number of effective drug therapies now available [21], including TTR protein stabilizers (tafamidis, diflunisal) [22–24], antisense oligonucleotide therapies (inotersen, eplontersen) [25], and RNA interference therapies (patisiran, vutrisiran) [26, 27]. If initiated early in the disease, these therapies can lead to a delay in progression, stabilization, or even a slight improvement in the disease [24]. However, although awareness of ATTRv has increased over recent years, establishing an early diagnosis remains a challenge and the rate of misdiagnosis is high. In addition, counselling is crucial to propose genetic testing of siblings and children, in order to detect carriers and early onset of the disease [28]. 

This study has several limitations. The main limitation is the fact that the questionnaire did not include a question on the number of doses of IVIg the patients had received. There could also be some possible recruitment bias, both at the HCP and patient level. The questionnaires were sent to 1100 HCPs, but although participation was voluntary and anonymous only 110 (10%) took part in the final survey. Thus, the HCPs recruited only represent a small sample of HCPs across France and the results cannot be generalized. Furthermore, because of this low level of recruitment, there was no direct comparison of the rate of *TTR* testing by HCPs employed in reference centres for neuromuscular disorders and those working outside these centres. Thus, our results are only an indication and may not truly represent ATTRv diagnosis in real-life.

The strength of the study is the fact that it was carried out in a large number (*n* = 108) of secondary care French hospitals and across all regions of France. The study also included a large number of patients with suspected CIDP.

## Conclusion

In this study, 13.7% of IVIg-NR suspected CIDP patients tested positive for a *TTR* mutation and were diagnosed with ATTRv. The most common reason for considering an alternative diagnosis of ATTRv was orthostatic hypertension. HCPs should recognize the need to carry out genetic *TTR* testing if a patient with suspected CIDP fails to respond to IVIg. The red flags for suspecting a diagnosis of ATTRv are discussed in the 2021 EAN/PNS guidelines [4, 5]. 

## Electronic supplementary material

Below is the link to the electronic supplementary material.

Supplementary Material 1

Supplementary Material 2

## Data Availability

All data supporting the findings of this study are available within the paper and its Supplementary material.
