# Supplementary material for: Diagnosis of hereditary transthyretin amyloidosis in patients with suspected chronic inflammatory demyelinating polyneuropathy unresponsive to intravenous immunoglobulins: results of a retrospective study
Source: Orphanet J Rare Dis. 2025 Mar 1;20:95. doi: 10.1186/s13023-025-03589-4 (PMC11871584; doi:10.1186/s13023-025-03589-4)
Supplement: Supplementary file 2 — Supplementary Material 2 [file 13023_2025_3589_MOESM2_ESM.docx]

**Appendix Questionnaire**

**Screening questions for clinicians**

1. ***What is your specialty?***

❑ Neurologist

❑ Intern

❑ Other 🡺 STOP

1. ***What is your type of practice?***

❑ Private 🡺 STOP

❑ Mixed: private and hospital

❑ Hospital

*If the type of practice is mixed or hospital*

1. ***Please specify the type of establishment in which you work***

❑ University Hospital ❑ CH/CHG ❑ Clinic ❑ CLCC

1. ***How many years have you been practicing?***

_____ If <3 years or >30 years 🡺 STOP

1. ***Pease specify the department where your practice is located***

Department: I__I__I

1. Ile de France: 75;77;78;91;92;93;94;95
2. Nord-Ouest: 14;18;27;28;36;37;41;45;50;61;76;16;17;22;29;35;44;49;53;56;72;79;85;86
3. Nord-Est: 2;8;10;21;51;52;58;60;71;80;89;59;62;25;39;54;55;57;67;68;70;88;90
4. Sud-Ouest: 9;12;19;23;24;31;32;33;40;46;47;64;65;81;82;87
5. Sud-Est: 4;5;6;11;13;20;30;34;48;66;83;84; 1;3;7;15;26;38;42;43;63;69;73;74
6. ***Please indicate your sex***

❑ Male

❑ Female

1. ***Please specify your age***

I__I__I years

1. <45 years
2. 45-54 years
3. 55-59 years
4. 60-69 years
5. ≥70 years

**Questionnaire**

1. **How many CIDP patients do you manage in your practice?**

I__I__I__I patients with CIDP **🡺If 0 STOP**

1. **Among these CIDP patients, how many are currently receiving treatment with intravenous IgG?**

I__I__I__I CIDP patients treated with IgG

1. **Among these patients treated with IgG, how many are currently non-responders to 3 or 4 months of IgG treatment?**

I__I__I__I non-responders to IgG

1. **Among these non-responders to IgG, how many have been prescribed a genetic test to look for ATTRv?**

I__I__I__I patients prescribed a genetic test for ATTRv

1. **If you sent these patients for a genetic test, which structure did you send them too?**

Open question: written response

1. **Are you aware of the existence of a reference center for familial amyloid neuropathies at the Hôpital Bicêtre?**

**❑ Yes**

**❑ No**

1. **Among these patients for whom a genetic test has been prescribed, how many patients have actually undergone a test (patients for whom you have received the results of the ATTRv test)?**

I__I__I__I patients who have undergone a genetic test for ATTRv

1. **Which structure did you send your patients to in order to get the genetic test for ATTRv?**
2. To the reference center for amyloid neuropathies at the Hôpital Bicêtre
3. To another structure, please specify which: ____________________
4. **Among these patients who have undergone a genetic test for ATTRv, how many tested positive?**

I__I__I__I patients positive for ATTRv

Question 10 should be answered for each positive patient

1. **What steps did you take with [patient X] whose genetic test for ATTRv was positive?**

|  | **Yes** | **No** | **If yes: specify** |
| --- | --- | --- | --- |
| 1. You referred them to the reference center for familial amyloid neuropathies at the Hôpital Bicêtre |  |  |  |
| 1. You referred them to a neuro-muscular reference center in your region |  |  | If yes: specify which  _____________________ |
| 1. You managed them yourself |  |  |  |
| 1. You carried out an investigation of their family |  |  |  |
